# Management of peri-prosthetic joint infection and severe bone loss after total hip arthroplasty using a long-stemmed cemented custom-made articulating spacer (CUMARS)

**DOI:** 10.1186/s12891-021-04237-1

**Published:** 2021-04-16

**Authors:** J. Quayle, A. Barakat, A. Klasan, A. Mittal, G. Chan, J. Gibbs, M. Edmondson, P. Stott

**Affiliations:** 1 Brighton and Sussex University Hospitals, Brighton, UK; 2 Department for Orthopaedics and Traumatology, Kepler University Hospital GmbH, Krankenhausstrasse 9, 4020 Linz, Austria; 3 Johannes Kepler University Linz, Altenberger Strasse 69, 4040 Linz, Austria

**Keywords:** Prothesis-related infections, Prothesis failure, Methylmethacrylate, Arthroplasty, Replacement, Hip, Femur, Bone loss, CUMARS

## Abstract

**Background:**

There is little evidence on techniques for management of peri-prosthetic infection (PJI) in the context of severe proximal femoral bone loss. Custom-made articulating spacers (CUMARS) utilising cemented femoral stems as spacers was described providing better bone support and longer survival compared to conventional articulating spacers. We retrospectively report our experience managing PJI by adaptation of this technique using long cemented femoral stems where bone loss precludes use of standard stems.

**Methods:**

Patients undergoing 1st stage revision for infected primary and revision THA using a cemented long stem (> 205 mm) and standard all-polyethylene acetabulum between 2011 and 2018 were identified. After excluding other causes of revision (fractures or aseptic loosening), Twenty-one patients remained out of total 721 revisions. Medical records were assessed for demographics, initial microbiological and operative treatment, complications, eradication of infection and subsequent operations. 2nd stage revision was undertaken in the presence of pain or subsidence.

**Results:**

Twenty-one patients underwent 1st stage revision with a cemented long femoral stem. Mean follow up was 3.9 years (range 1.7–7.2). Infection was eradicated in 15 (71.4%) patients. Two patients (9.5%) required repeat 1st stage and subsequently cleared their infection. Three patients (14.3%) had chronic infection and are on long term suppressive antibiotics. One patient (4.8%) was lost to follow up before 2 years. Complications occurred in seven patients (33%) during or after 1st stage revision. Where infection was cleared, 2nd stage revision was undertaken in 12 patients (76.5%) at average of 9 months post 1st stage. Five (23.8%) CUMARS constructs remained in-situ at an average of 3.8 years post-op (range 2.6–5.1).

**Conclusions:**

Our technique can be used in the most taxing of reconstructive scenarios allowing mobility, local antibiotic delivery, maintenance of leg length and preserves bone and soft tissue, factors not afforded by alternative spacer options.

## Background

Managing peri-prosthetic joint infection (PJI) is difficult with high numbers of failures, complications [1] and clinical outcomes comparable to or worse than revision surgery [2, 3]. The presence of significant bone loss on the femoral or acetabular side, amplifies these challenges. Given the rise in the number of joint replacements performed [4, 5], a commensurate increase in the number of PJI is anticipated. Furthermore, as patients are living longer and requiring multiple revision joint surgery, treating PJI where there is already extensive bone loss due to stress shielding, osteolysis or implant loosening will also increase [6]. For this reason, successful strategies for treating infection in this setting are required.

Where bone loss is extensive, infections are chronic or recurrent and hips have previously been operated on repeatedly then debridement and implant retention (DAIR) [7] is not an option. Therefore, one or two-stage exchange is required. This involves debridement of the infected tissues, removal of the implants followed by reconstruction immediately (one stage) or after a delay (two stage). While two stage revision is seen as the gold standard treatment for PJI due to the high rates of infection eradication, there is increasing evidence to challenge this point of view [8–11].

In two-stage revision, a temporary spacer is typically used. These are split into two types: non-articulating spacers e.g. cement block or beads, or articulating spacers made commercially or fashioned intra-operatively. While the benefits of the spacer are multiple [12–15], there are a several potential spacers complications such as fracture and dislocation which are even more common where they are not supported by host bone [14–16]. Commercial spacers do not come in sufficient lengths to provide stability in patients with bone loss.

Non-articulating spacers can be made with cement only, or a combination of cement and metalwork - examples include intramedullary nails or Kirschner wires [17, 18]. Whilst these can support a damaged bone better, they do not allow the hip to articulate. This causes the patient pain and disability and makes implanting the second stage more difficult.

The Custom-Made Articulating Spacer (CUMARS) [19] consists of a standard all polyethylene acetabular component and Exeter Universal stem (Stryker Orthopaedics, Mahwah, New Jersey) implanted with antibiotic loaded cement optimised to the relevant organism. It has been used successfully in the management of PJI. However, this technique is not suitable in the context of extensive femoral bone loss due to the high risk of peri-prosthetic fracture. To adapt the technique, our institution utilised the Exeter V40 Long (≥ 205 mm) Femoral stems (Stryker). This allowed femoral defects to be bypassed, ensured an adequate cement mantle and maintained leg length and function. This is the first published description of this technique and one of the largest series of patients treated for PJI in the context of severe femoral bone loss.

## Methods

Patients treated with the CUMARS technique and Exeter stems ≥205 mm were identified from our retrospective hospital database, theatre logbooks and arthroplasty infection records. All patients were treated at a single tertiary referral centre by fellowship trained arthroplasty surgeons (PS, JG, ME). Where possible, a pre-operative microbiology diagnosis was made with joint aspiration or blood culture. The International Consensus Meeting on peri-prosthetic Joint Infection criteria [9] were used to confirm the presence of infection. A surgical and microbiology strategy was selected at a pre-operative meeting. Copal G + C (Heraeus Medical, Newbury, United Kingdom) cement was used in all but one case in which Simplex P (Howmedica, Limerick, Ireland) cement was chosen. Vancomycin and/or meropenem were added to the cement up to the maximum allowable dose in accordance with the manufacturer’s guidelines.

Each procedure was performed via a posterior approach using the old scar where feasible. Fluid was aspirated from the joint prior to arthrotomy and assessed for cell count, gram stain and culture. Five or more tissue samples were subsequently taken for microbiology culture using clean instruments. Antibiotics were given after collection of adequate samples. Bone loss was avoided accepting for the necessity of implant extraction and adequate debridement. Where necessary an extended trochanteric osteotomy (ETO) was performed to facilitate extraction of the femoral implant. Thorough debridement and lavage was then undertaken.

**Fig. 1:**
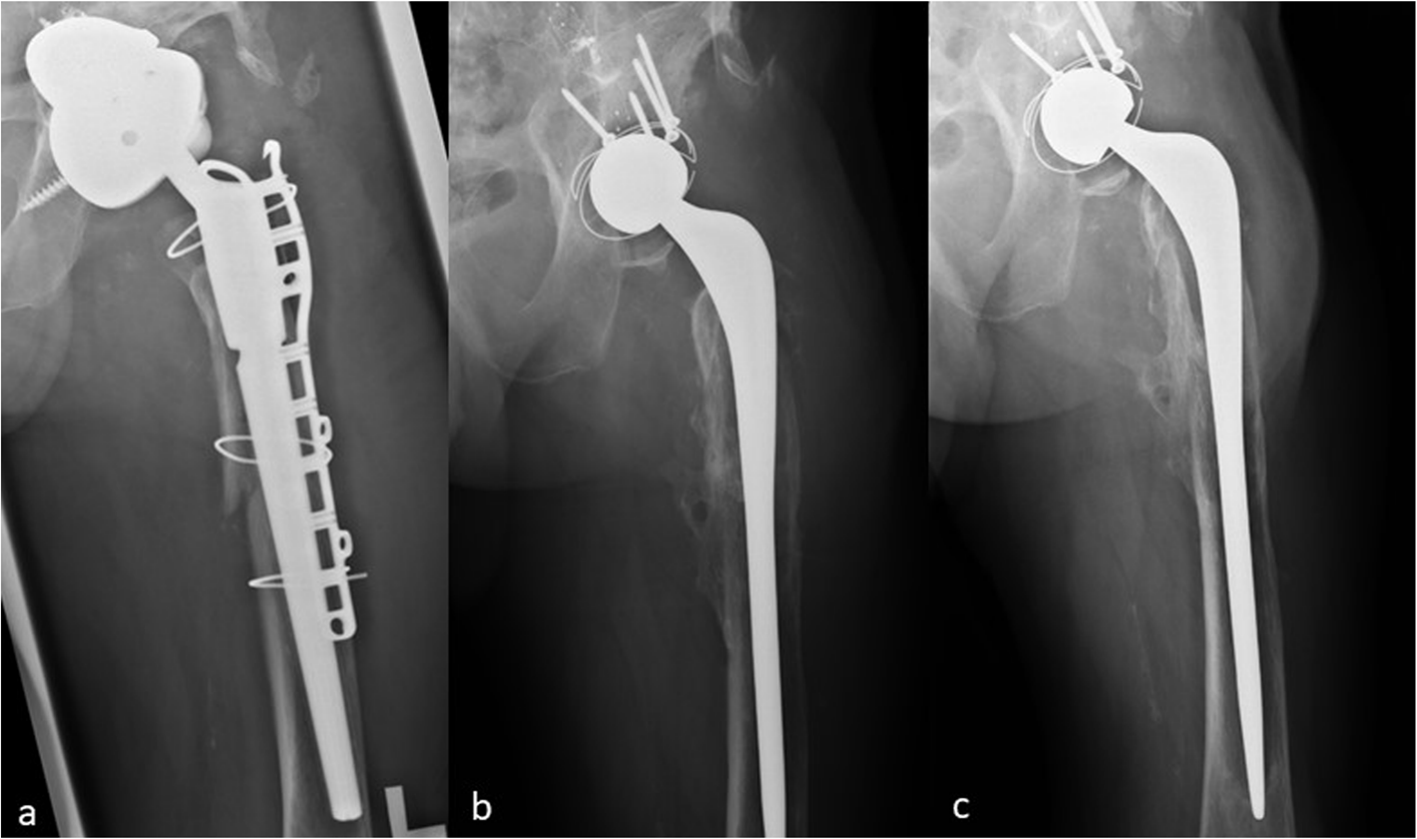
**a** Pre-operative Anteroposterior (AP) radiograph showing a modular fluted revision femoral stem and cup-cage acetabular construct which were proven to be infected by tissue diagnosis (**b**) AP radiograph after 1 year of explantation of infected prothesis and treatment with a long CUMAR technique (**c**) AP radiograph 4.5 years post-operatively demonstrating continued survival of the 1st stage with complete eradication of the infection

Bone stock was assessed and an Exeter Stem long enough to bypass the bony defect and achieve adequate fixation was selected (range 205-260 mm). This was implanted with cement that had been modified by the addition of antibiotics. The cement was either hand packed or injected into the femoral canal and directly applied to the implant. Finger pressurisation was used upon stem insertion. Acetabulum reconstruction was undertaken using a size F Trident insert (after burring the back to gain stability within the cement mantle), a RimFit cup or the Exeter Contemporary flanged cup (all Stryker Orthopedics). To maximise stability, a 36 mm inner diameter was chosen. In the presence of severe acetabular bone loss, screws and wires were inserted into the pelvis to act as a scaffold to ensure adequacy of the cement mantle. Post-operative intravenous and/or oral antibiotics were given for a minimum of 6 weeks (Fig. 1). Patients were allowed to weight bear as tolerated post-operatively.

The post-operative period was monitored with serial C-Reactive Protein (CRP) and white cell count measurements. Patients were typically reviewed at 6 weeks, 3 months, 6 months and 1 year. Patient were assessed clinically using the Oxford Hip Score (OHS). The second stage was undertaken in the presence of ongoing pain or radiological evidence of subsidence along with normal inflammatory markers. A lower threshold was applied for patients with concomitant high-grade acetabular defects. Infection was considered eradicated if there is normalisation of inflammatory markers, no clinical failure (healed wound without fistula or drainage and painless joint, no subsequent surgical intervention owing to infection, and no death caused by a condition directly linked to PJI including sepsis, necrotizing fasciitis) were present at a minimum follow up of 2 years in accordance with the International Consensus Meeting Delphi criteria [10, 11].

Femoral and acetabular bone stock were assessed on the basis of pre-operative radiographs by the authors (AB, JQ) and classified according to the Paprosky classification [12]. The assessment was undertaken separately and where disagreement existed, one of the senior authors (PS) took the final decision. This was cross referenced against intra-operative findings.

Consecutive patients treated for THA PJI with the long CUMARS technique between November 2011 and July 2018 were included in our analysis. This was the only technique used for patients whose femur would not support a standard-length cemented stem during the study period. Follow up length was considered as the period from date of 1st stage through to most recent clinical assessment or death. Primary outcome data such as clearance of infection (as reflected by normalisation of inflammatory markers), revision of 1st stage (for persistence or recurrence of PJI), 2nd stage revision (for pain or subsidence) as well as secondary outcomes such as intra-operative or post-operative non infection related complications were available for all patients. A power analysis was not performed as all cases were included in the study. Normally distributed data are presented as mean ± standard deviation (SD). Statistical analysis was performed using SPSS 24 (IBM, Armonk, NY, U.S.).

## Results

### Demographics

Our study identified 21 patients that met the inclusion criteria. The mean age was 72.4 years (± 6.1). This included eight males and 13 females. Reasons for revision are documented in Table 1. The majority of patients (15) had chronic infections. However, there was also one early acute, three late acute and two recurrent PJI infections (Table 2). Details of the primary operations were available in 17 patients. The infected implants had been in-situ for an average of 6.5 years (range 0.1–20.4 years) at the time of 1st stage revision.

**Table 1:** Reason for Revision

Reason for Revision	Number (%)
Infected Primary THA	9 (42.9%)
Infected Primary Revision THA	11 (52.4%)
Infected Hemiarthroplasty	1 (4.8%)

**Table 2:** Infection Presentation type

Presentation Type	Number (%)
Early Acute (< 3 month)	1 (4.8%)
Late Acute (3 month – 12 month)	3 (14.3%)
Chronic (> 12 month)	15 (71.4%)
Recurrent	2 (9.5%)

The most common organism cultured was coagulase negative staphylococcus (CNS) with *Staphylococcus aureus* and mixed growth being the next most common (Table 3). There was one patient where there was no growth. They were revised on clinical and radiological suspicion of PJI due to presence of persistent hip pain, elevated inflammatory markers and aspiration of pus from around the THA.

**Table 3:** Microbiology Results

Organism	Number (%)
No growth	1 (4.8%)
Staph. Aureus	3 (14.3%)
Coagulase Negative Staph	9 (42.9%)
Streptococcus	1 (4.8%)
E.coli	2 (9.5%)
Mixed	3 (14.3%)
Bacteroides	1 (4.8%)
Citrobacter	1 (4.8%)

Femoral bone loss is described in Table 4, one patient was classified as Type II (a long stem was used to bypass an anterior perforation of the femoral canal), 15 patients had Type IIIA and 4 patients had Type IIIB femurs. An ETO to allow stem removal was required in 13 patients. Acetabular bone loss is described in Table 5 with 11 patients classified as type I, six patients as Type II (1 2A, 4 2B, 1 2C) and four patients as Type 3 (3 IIIA, 1 IIIB).

**Table 4:** Femoral Bone Loss (Paprosky Classification)

Type	Number/percentage of patients
I	0 (0%)
II	1 (4.8%)
IIIA	16 (76.2%)
IIIB	4 (19.0%)

**Table 5:** Acetabular Bone Loss (Paprosky Classification)

Type	Number/percentage of patients
I	11 (52.4%)
IIA	1 (4.8%)
IIB	4 (19.0%)
IIC	1 (4.8%)
IIIA	3 (14.3%)
IIIB	1 (4.8%)
IV	0 (0%)
V	0 (0%)

### Eradication of infection

**Fig. 2:**
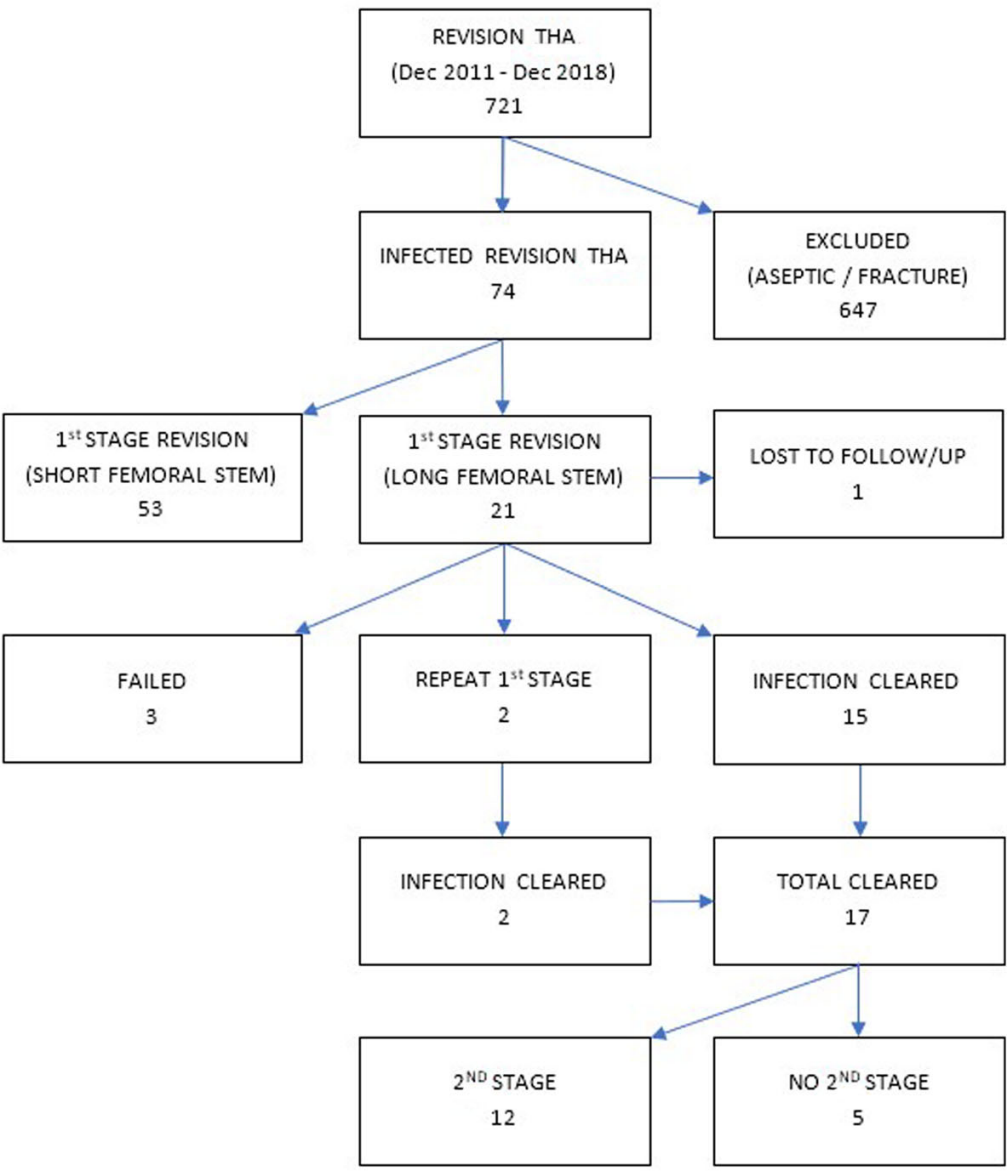
Selection flow chart

Of the 21 patients treated with CUMARS, 15 patients (71.4%) met the criteria for eradication of infection. The average follow up in this group was 3.9 years (range 1.7–7.2). There were two patients who had repeat 1st stage and subsequently cleared the infection meaning 81.0% of all cases were infection free at final follow up. Of those not meeting the criteria for eradication of infection, there was one patient who died within the minimum follow up period from causes unrelated to PJI, 6 months post-operatively. A further three patients are maintained on suppressive antibiotics (Fig. 2).

### Complications

There was seven (33.3%) complications (Table 6) excluding recurrent infection. These included 2 dislocations following 1st stage revision, both in patients who had non-unions of their ETO. Neither patient required an expedited 2nd stage, though one required a dual mobility cup at 2nd stage. There were three intra-operative fractures. Of these, two were of the greater trochanter despite performing an ETO. One was managed with cables, the other required a plate. There was two post-operative fractures of the distal femur which required open reduction and internal fixation.

**Table 6:** Complications

Complication	Number (%)
Intra-operative Fracture	3 (14%)
Post-operative Fracture	2 (9.5%)
Dislocation	2 (9.5%)

### Mortality

There were two deaths (9.5%) during the study period, with one occurring before 2 years. Of these, both died of causes unrelated to PJI.

### Clinical outcomes

In patients with a minimum 2 year follow up and where infection was eradicated, the average time from 1st stage to end of the review period or death was 3.9 years, ranging from 2.0 to 7.2 years. Of the 17 cases with eradication of infection, 12 (70.5%) have had 2nd stage revision, with one of those occurring after repeat 1st stage. The time between stages was on average 35.8 weeks (range 15.9–72 weeks). Patient reported outcome scores were available on 75% of patients who underwent 2nd stage revision with an average OHS of 25.1 at final follow up (range 7–48). This compares to an OHS of 25.8 in the 5 patients who only had 1st stage revision. This difference did not achieve statistical significance.

### Unplanned revisions

As regards the 12 patients who had 2nd stage revision, none had a planned re-revision due to recurrence of infection or aseptic loosening at the latest follow-up. One patient had an unplanned revision of their acetabulum at 6 weeks following 2nd stage revision due to recurrent dislocation. This patient has had no further issues at 6.2 years following this latest surgery.

## Discussion

The successful management of PJI is challenging. In the presence of significant bone loss, these difficulties are multiplied. This adaptation of the CUMARS technique has the versatility to be used as an interim spacer and in some patients as a single stage procedure. This is achieved through a stable cemented construct that preserves bone stock, prevents soft tissue contracture and allows unrestricted mobility. This is evidenced by the extended period between 1st and 2nd stage revision. This is not possible using alternative techniques which have limited rotational and longitudinal stability [15, 20].

Given the small numbers of patients who suffer this complication, the literature on the treatment of PJI in the context of severe proximal femoral bone loss is scarce. Multiple techniques have been described which include the use of cement loaded Kuntscher nails [21], modular articulating antibiotic spacers [22], long stem uncemented THA with antibiotic impregnated allograft bone [23] and endoprostheses [1]. Despite the multitude of surgical descriptions, there are only four comparable case series of more than five patients identified in our literature search [17, 18, 24]. Our study is the largest to report on spacer design in the presence of femoral deficiency with one of the longest follow-ups.

Ben-Lulu et al. [18] report the use of a femoral antibiotic-impregnated cement spacer mould with a metal endoskeleton that was press fit into a pre-cut reamed intramedullary nail in 11 patients. Patients were allowed to touch-weight bear between 1st and 2nd stage procedures. Infection was eradicated in 90.9% but after only 1.25 years (range 1–1.5 years). Reimplantation was performed on average 3.5 months post 1st stage revision. Excluding death and recurrence of infection, they had two complications (18.2%): one dislocation and one dissociation between the nail and the spacer.

Hsieh et al. [17] report the outcomes of 8 patients with femoral deficiencies and one with a combined acetabular and femoral deficiency at 4.2 years follow up. They used a custom-made articulating spacer reinforced with Kirschner wires. Patients could touch-weight bear until 2nd stage revision at an average of 3.2 months. They had three spacer related complications including two spacer fractures and a dislocation.

Winkler et al. [23] treated 37 patients with PJI using uncemented prostheses and antibiotic impregnated bone allograft. Some had extensive femoral bone loss and were given long stems but there was no further analysis of this subset. Overall a high level of infection eradication (92%) was observed. Similar to our study, they advocate this as a single stage procedure with an early repeat 1st stage revision where this fails. The technique is not advised in the presence of Paprosky type 3 acetabular defects.

Finally, in two papers by Alvand and Grammatpolous [1, 24] they report on endoprosthetic replacements used in treating PJI in 40 hip patients with a 5 year follow up. They found an overall infection eradication of 82.5%. Their overall complication rate excluding recurrence and death was 40% however it is unclear which were due to the spacer. The time between 1st and 2nd stage procedures isn’t reported, though the 2nd stage appears to be mandatory in the majority of cases (70%). A proportion (30%) had a planned single stage due to frailty or because the bone stock prohibited formal two stage revision.

This technique has multiple advantages and has been demonstrated successfully in a case-series of 53 patients who have underwent first stage revision using standard primary stems [25]. Firstly, it uses readily available materials that are familiar to surgeons who cement their prostheses without the need to extend the indications of an implant e.g. femoral nail or spend large amounts on endoprostheses. The financial cost to the patient and the hospital of PJI is huge. Vanhegan [26] and Kapadia [27] estimate the difference in cost between a septic revision of infected THA and either an aseptic revision or a primary THA to be £10,000 and $63,000 respectively. Hence, managing the financial burden through the use of more cost-effective implants is imperative.

Secondly, the use of cement allows delivery of high levels of localised antibiotics that can be tailored to the appropriate cultured microbiology [12, 27] and avoid the complications associated of systemic administration [12]. The use of antibiotic loaded cement is part of the gold standard for treating PJI [28].

Thirdly, all patients were allowed to weight bear as tolerated. This functions to maintain bone stock, muscle mass and avoids complications of immobility. Allowing patients greater levels of independence and function is desirable both to facilitate rehabilitation and to reduce the burden on social care.

Fourthly, patients requiring this technique have histories littered with multiple revision surgeries, chronic infections, significant co-morbidities and bone loss. Despite these challenges, this technique achieves high levels of infection eradication comparable to current published literature [1, 17, 18].

Fifthly, this technique preserves overall bone stock. While complications and infection clearance are comparable with those achieved using endoprosthetic replacement, it does maintain options for future revision.

Finally, while the majority of patients have a 2nd stage procedure, it is not mandatory. In our cohort, there was on average 9 months between stages and five patients have cleared infection but have not undergone 2nd stage revision. These implants have survived an average of 3.8 years.

Like many of the papers addressing PJI, it has a number of weaknesses. Firstly, this is a retrospective review although the data are predominantly complete. Secondly, it is a heterogenous group of patients with a variety of presentations. There are also a vast range of primary implant histories. Thirdly, functional scoring was not available for all patients but the comparable eradication and revision rate in similar series serve as a proxy. This underpowered the clinical outcomes analysis, though there was a clear trend in all three measures. These case series are small and only multicentric studies performing the same technique would achieve sufficient statistical power. Fourthly, the acetabular defects and the cups used were different which may affect the results. This demonstrates the versatility of the technique.

## Conclusion

We report the results of a technique that can be used in the most taxing of reconstructive scenarios. The use of cemented long stem implants allows mobility, local antibiotic delivery, maintenance of leg length and preserves bone and soft tissue, factors not afforded by alternative spacer options. It achieves eradication of infection in a high proportion of patients. 2nd stage revision can be delayed and, in some patients, deferred indefinitely.

## Data Availability

The datasets used and/or analysed during the current study are available from the corresponding author on reasonable request.

## Abbreviations

CRP: C-Reactive Protein
CUMARS: Custom-Made Articulating Spacer
DAIR: Debridement, Antibiotics, Implant Retention
ETO: Extended Trochanteric Osteotomy
PJI: Peri-prosthetic Joint Infection
PROSTALAC: Prosthesis with Antibiotic Loaded Acrylic Cement
THA: Total Hip Arthroplasty
